# Assessment of chronic aortic regurgitation using end-diastolic flow reversal in the upper descending aorta: diagnostic accuracy and prediction of aortic valve surgery in a prospective echocardiography and cardiac magnetic resonance imaging study

**DOI:** 10.1186/s44156-025-00101-3

**Published:** 2026-01-05

**Authors:** Knoll Katharina, Stefanie Rosner, Stefan Gross, Hasema Persch, Daniel Braun, Martin Orban, Wibke Reinhard, Martin Hadamitzky, Carolin Sonne

**Affiliations:** 1 Department of Cardiology, School of Medicine and Health, German Heart Centre Munich, TUM University Hospital, Technical University of Munich, Munich, Germany; 2 DZHK (German Centre for Cardiovascular Research), Partner Site Munich Heart Alliance, Munich, Germany; 3 Department of Internal Medicine B, University Medicine Greifswald, 17475 Greifswald, Germany; 4 DZHK (German Centre for Cardiovascular Research), Partner Site Greifswald, 17475 Greifswald, Germany; 5 Division of Sport and Rehabilitation Medicine, Center of Internal Medicine, Ulm University Hospital, Leimgrubenweg 14, 89075 Ulm, Germany; 6 Department of Medicine I, Ludwig-Maximilians University, Marchioninistr. 15, 81377 Munich, Germany; 7 Department of Radiology, German Heart Center of Munich, Technical University of Munich, Lazarettstr. 36, 80636 Munich, Germany

**Keywords:** Aortic regurgitation, End diastolic flow reversal in the upper descending aorta (EFR), Multimodality imaging, CMRI, Echocardiography, Aortic valve surgery

## Abstract

**Background:**

This study aimed to assess the utility of end-diastolic flow reversal in the upper descending aorta (EFR) for echocardiographic grading of aortic regurgitation (AR) severity, using cardiac magnetic resonance imaging (CMRI) as the reference. Additionally, we evaluated the role of EFR in predicting the need for aortic valve (AV) surgery during mid-term follow-up.

**Methods:**

Sixty-six patients (mean age 53 ± 15.0 years, 73% men) underwent echocardiographic assessment, including (semi-)quantitative parameters such as proximal isovelocity surface area (PISA), effective regurgitation orifice area (EROA), AR volume (AR-Vol), vena contracta (VC) and EFR, compared against CMR-derived regurgitant fraction (RF). Multivariable regression analysis was applied evaluating predictors for severe AR and AV surgery.

**Results:**

According to CMRI, 13 patients had no, 16 mild, 18 moderate and 19 severe AR. All echocardiographic parameters demonstrated good diagnostic accuracy (AUC: EFR 0.84, VC 0.82, PISA 0.84, EROA 0.83, AR-Vol 0.86; all *p* < 0.001). Logistic regression identified EFR (*p* = 0.022) and VC (*p* = 0. 015) as significant predictors of severe AR. During 62 months follow-up, 16 of 53 patients (24%) underwent AV surgery. All echocardiographic parameters, except PHT, were significantly different between patients receiving AV surgery compared to patients without surgery (PISA, EROA, AR-Vol and VC *p* < 0.001, EFR *p* = 0.043). However, VC remained the only parameter significantly associated with time to AV surgery (Cox-regression analysis, *p* = 0.024).

**Conclusions:**

EFR is a robust and reliable parameter for AR assessment, particularly in distinguishing moderate from severe AR, and should be incorporated into comprehensive echocardiographic assessment. However, VC demonstrated stronger prognostic relevance for predicting surgical intervention.

**Supplementary Information:**

The online version contains supplementary material available at 10.1186/s44156-025-00101-3.

## Background

Echocardiography is the primary imaging modality for the assessment of aortic regurgitation (AR) severity and to identify its cause, as it is almost universally available, cost-effective, non-invasive and easily repeatable for the longitudinal evaluation of patients with chronic AR. Under optimal examination conditions, the indication for surgery in severe AR can be provided exclusively based on symptoms and echocardiographic measurements [1]. However, quantification of AR can be extremely challenging at times, despite the integrated use of multiple (semi-)quantitative echocardiographic parameters, due to feasibility and reproducibility issues [5–7]. The European Association of Cardiovascular Imaging (EACVI) and the American Society of Echocardiography (ASE), as well as the British Society of Echocardiography (BSE) [2–4] recommend using an integrated approach with multiple (semi-)quantitative echocardiographic parameters. When echocardiography is inconclusive, current ESC guidelines recommend cardiac magnetic resonance imaging (CMRI) for additional quantification of AR [5].

As an adjunct examination method for AR, CMRI offers high accuracy for volume and flow quantifications [6]. It enables direct quantification of ante- and retrograde blood flow across heart valves using specialized flow-sensitive sequences, thus facilitating the quantification of regurgitant volume and regurgitant fraction (RF) [7–12].

In echocardiography, end diastolic flow reversal in the upper descending aorta (EFR) is an AR grading parameter measured distant from the aortic valve (AV). This measurement is easily performed from the suprasternal view and avoids the limitations associated with measurements at the AV level, such as poor acoustic windows, shadowing due to calcification, jet eccentricity and multiple jets. An EFR threshold of >18 cm/s predicted severe AR in the original study by Tribouilloy et al. [13]. The EACVI, ASE and BSE recommend the inclusion of EFR-measurement in AR severity stratification [3, 14], as EFR-velocity has been shown to be reliably increased in severe AR [13, 15–17]. Yet it remains unclear whether this parameter improves the diagnostic accuracy of echocardiographic grading of AR and the prediction of need for AV surgery. The aims of our study are: (1) to assess the utility of EFR in echocardiographic grading of AR severity using CMRI as a reference, and (2) its potential as predictor for the need of AV surgery on mid-term follow-up.

## Methods

This prospective, single-center study (German Heart Center Munich, Germany) assessed the value of echocardiographic parameters for grading of aortic regurgitation (AR) in 66 patients between August 2012 and April 2017. The study was approved by the ethics committee of the Technical University of Munich (TUM), faculty of medicine, and was conducted in accordance with the principles outlined in the Declaration of Helsinki. All participants provided informed consent to participate in the study.

Of the 66 patients who underwent assessment with cardiac magnetic resonance imaging (CMRI) and echocardiography, 53 patients had chronic AR, while 13 patients without AR, confirmed by both echocardiography and CMR, served as controls and were consecutively recruited from patients undergoing CMR for other indications.

Examiners of both the CMRI and echocardiography studies were blinded regarding AR grading in the corresponding examinations. AR grading for statistical analyses was determined by calculating the AR-regurgitation fraction (RF) in CMRI examinations. Exclusion criteria were contraindications for CMRI, absence of echocardiographic EFR-measurement, any heart rhythm other than sinus rhythm, previous cardiac surgeries, severe concomitant valvular diseases, severe cardiac abnormalities or diseases except coronary artery disease (e.g. hypertrophic cardiomyopathy, cardiac amyloidosis), hemodynamic instability, age under 18 years, pregnancy, and morbid obesity (body mass index > 40 kg/m2).

### Echocardiography

Echocardiographic measurements were performed by an experienced senior fellow or an attending cardiologist, using commercially available echocardiographic machines (Philips iE33 and EPIQ 7G, Philips Medical Systems, Andover, MA). Assessment of AR severity was performed according to EACVI Guidelines [2].

**Fig. 1:**
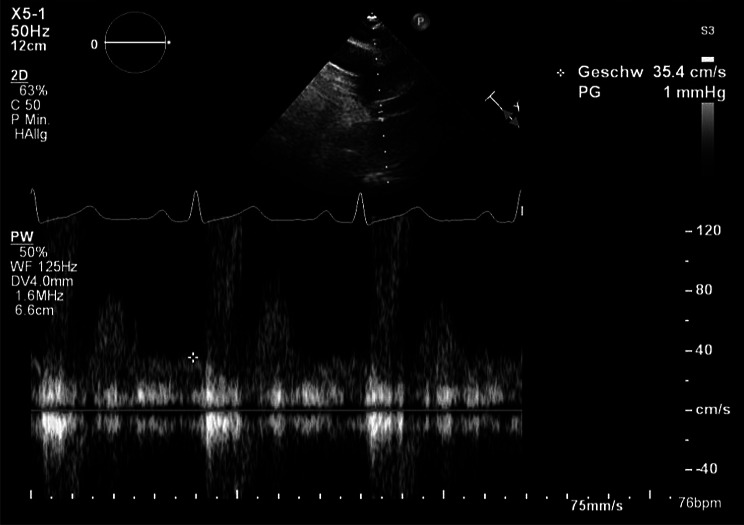
Measurement of end-diastolic flow reversal in the upper descending aorta. Positioning of the probe in the descending aorta just beneath the origin of the left subclavian artery using a suprasternal notch view. End-diastolic flow reversal (EFR) was determined at the peak R wave on a simultaneously recorded electrocardiogram using pulsed wave Doppler

EFR was obtained in all 66 patients by pulsed-wave Doppler in end-diastole, just beneath the origin of the left subclavian artery using a suprasternal notch view. The Doppler beam was aligned in parallel with the axis of aortic blood flow to show a maximal systolic (negative) flow velocity curve. Doppler filters were adjusted to minimal values, and gains were reduced to obtain a contrasted but uninterrupted display of the Doppler frequencies. The end diastolic flow velocity (cm/s) was determined at the peak R wave on a simultaneously recorded electrocardiogram (Fig. 1). Three EFR-measurements were taken per patient in all patients: two consecutive measurements by one examiner and one additional measurement by a second blinded examiner, for assessing intra- and inter-observer variability. The initial value measured by the first observer was used for analysis.

The following additional recommended echocardiographic measurements were obtained. The proximal isovelocity surface area (PISA) was measured using continuous-wave color Doppler. The flow convergence zone was visualized from the apical view in cases with central jets and from the parasternal long-axis view in cases with eccentric jets, in order to align the flow convergence with the ultrasound beam. A large zoom of the flow convergence zone was obtained, and the baseline velocity was shifted in the direction of the regurgitant flow until the region of flow convergence was clearly visualized with an optimal hemispheric shape. The radius (r) of the flow convergence was measured between the first aliasing contour and the aortic regurgitant orifice in early diastole, at the same time as the peak regurgitant velocity. The effective regurgitation orifice area (AR-EROA) was calculated as the flow velocity (2πr² multiplied by aliasing velocity) divided by the peak velocity of the regurgitant jet, as recorded by continuous-wave Doppler. The regurgitant volume (AR-Vol) was calculated as the product of the EROA and the time-velocity integral (TVI). The vena contracta (VC), defined as the narrowest portion of the regurgitant jet downstream from the regurgitant orifice, was measured perpendicular to the jet. The aortic regurgitation jet pressure half time (PHT) was measured using continuous-wave Doppler, carefully aligned with the AR jet as visualized in color Doppler in the most appropriate view. Lastly, the left ventricular end-diastolic and end-systolic volumes (LVEDV and LVESV) were obtained to calculate the ejection fraction of the left ventricle (LV-EF), expressed as a percentage, using the biplanar Simpson method [18].

Recommended (semi-)quantitative thresholds for severe AR were used according to EACVI guidelines [11]: EROA ≥ 30 mm², AR-Vol ≥ 60 ml, AR-RF ≥ 50%, VC >6 mm, PHT < 200 ms, and EFR >18 cm/s. All executed AR measurements contributed to the overall clinical severity grading of AR in echocardiography.

### CMR procedure

CMR regurgitant fraction was used as the primary reference for AR severity, in line with guideline recommendations when echocardiography is inconclusive.

All CMRI measurements were performed by an attending of radiology, using a 1.5 T Scanner (Siemens Avanto, Siemens Healthineers, Erlangen) and dedicated phased-array cardiac coils during free breathing. Through-plane flow-sensitive gradient echo sequences at the sinu-tubular junction of the aortic root with retrospective gating were used (TR 29.5 ms, TE 2.8 ms, field of view 320 × 240 mm, slice thickness 5 mm, in plane resolution 1.25 × 1.25 mm, v_enc_ 150 cm/s typically, 2 segments typically, 3 repetitions). Flow volume was integrated over 30 measurements during one heart cycle and aortic RF was defined as total retrograde flow divided by total anterograde flow. AR-grade was classified by aortic RF, with thresholds based on established standards: 1–19% in mild, 20–39% in moderate and >40% in severe AR [11]. CMR was performed as part of a prospective research protocol, but imaging reports were available to treating clinicians. The imaging interval between echocardiography and CMR was less than 14 days in all patients.

### Clinical endpoints

Follow-up information was obtained by clinical visits or telephone contact. Verification of all stated events was ensured by hospital records or confirmation by the attending physician. The clinical endpoint of the study, AV surgery, included valve replacement as well as valve reconstruction. Decisions regarding aortic valve surgery were at the discretion of the attending physician, primarily guided by disease severity, imaging and clinical parameters, in line with clinical practice.

### Statistical analysis

Statistical analysis was performed using SPSS (IBM SPSS Statistics, version 22.0) and STATA (Version 18.5). A p-value < 0.05 was considered statistically significant. Data are presented as mean ± standard deviation (SD) or median (interquartile range) for continuous variables, as appropriate, and as frequencies (percentages) for categorical variables. Differences in baseline characteristics according to AR grade were tested using the Pearson χ^2^ test for categorical and an analysis of variance (ANOVA) for continuous variables. Differences in mean values of echocardiographic grading parameters (used as continuous variables) between AR severity grades as assessed by CMRI were tested by ANOVA, including pairwise post-hoc group comparisons (Tukey test). For EFR-velocity sensitivity, intra- and inter-observer agreement was assessed by calculating the intra-class correlation coefficient.

The overall echocardiographic grading of AR was compared to AR assessment by CMRI trough correlation analysis and Pearsons correlation coefficient. The diagnostic value of each (semi-)quantitative parameter for grading of AR was analyzed by calculating sensitivity and specificity and by ROC analysis. Sensitivity, specificity, positive predictive value (PPV), and negative predictive value (NPV) were determined for all echocardiographic parameters using the established cut-offs. The area under the receiver operating characteristics (ROC) curve (AUC) were calculated for the echocardiographic parameters using both the original and z-transformed values. Pairwise comparison if AUC values was performed using χ^2^ test.

Finally, a multivariable logistic regression model was used, including all echocardiographic parameters except for PHT, with a stepwise backward selection procedure and a conservative threshold of *p* = 0.157 for retaining variables in the model. This threshold is equivalent to the AIC criterion for selection of predictors [19]. The parameter PHT was excluded in the multivariable model, as PHT could only be measured in the presence of AR.

To assess the diagnostic relevance of the echocardiographic AR parameters, we compared patients needing AV surgery with those not needing surgery by Mann-Whitney-U-or Pearson χ^2^ test. Continuous variables were either z-transformed values or categorised using the established cut-off values. Time to event (AV surgery analysis) done calculating Kaplan-Meier survival curves for patients according to AR severity as graded by the individual echocardiographic parameters. Differences in Kaplan-Meier curves were evaluated using the Log-Rank test for differences. A multivariable Cox-Regression with a backward selection procedure and the same threshold as the logistic regression model was also calculated. For the Cox-Regression, the same echocardiographic parameters were used as for the multivariable logistic regression, and calculation of the Schoenfeld residuals revealed no violation of the proportional-hazards assumptions.

## Results

### Study cohort and baseline characteristics

Sixty-six patients were enrolled in this study, including 53 patients with mild to severe chronic AR based on CMRI, where AR severity was assessed using the AR-fraction. These patients were classified as follows: 16 with mild AR (AR1), 18 with moderate AR (AR2) and 19 with severe AR (AR3). Additionally, 13 patients without AR served as the control group (AR0).

Among the 53 patients with AR, the underlying cause was attributed to aortic dilation in 17 patients (32%), bicuspid AV morphology in 27 (51%), degenerative AV disease in 3 (6%), and flail of one of the aortic cusps in 2 patients (4%), while in one patient, the cause remained unclear. None of the patients had concomitant severe valvular disease of other heart valves.

Baseline characteristics of the entire cohort and according to AR severity are shown in Table 1. The mean age was 53 ± 15 years (range: 26–84 years), and 73% of the patients were male. Patients with AR were significantly older and had a higher prevalence of hypertension, aortic root dilatation and bicuspid AV morphology. The mean LV-EF, as assessed by both echocardiography and CMRI, was normal (> 55%) across all AR severity groups (Table 2). While left ventricular end-diastolic diameter (LVEDD) measured by echocardiography, and LV volumes assessed by CMRI, showed significant differences between AR severity grades, no statistically significant differences were observed in left ventricular end-systolic diameter (LVESD) as measured by echocardiography (Table 2).

**Table 1:** Baseline characteristics according to AR grade as assessed by CMRI

	AR0 (*n* = 13)	AR1 (*n* = 16)	AR2 (*n* = 18)	AR3 (*n* = 19)	total (*n* = 66)	*p*-value
Age (years)	42 ± 13	56 ± 12	50 ± 16	59 ± 13	53 ± 15	0.006
Male	6 (46%)	12 (75%)	13 (72%)	17 (89%)	48 (73%)	0.061
Bicuspid AV	0 (0%)	6 (38%)	13 (72%)	8 (42%)	27 (41%)	< 0.01
Ascending aorta > 40 mm	0 (0%)	10 (62%)	6 (33%)	8 (42%)	24 (37%)	0.008
Mild to moderate mitral insufficiency	0 (0%)	3 (19%)	7 (39%)	5 (26%)	15 (23%)	0.290
Diabetes mellitus	1 (8%)	0 (0%)	0 (0%)	1 (5%)	2 (3%)	0.504
Coronary artery disease	1 (8%)	3 (19%)	2 (11%)	1 (5%)	7 (11%)	0.612
(Prior) smoking	1 (8%)	4 (25%)	1 (6%)	1 (5%)	7 (11%)	0.198
Arterial hypertension	2 (15%)	9 (56%)	12 (67%)	10 (53%)	33 (50%)	0.036
Hyperlipidemia	1 (8%)	5 (31%)	8 (44%)	9 (470%)	23 (35%)	0.096
NYHA class I	12	12	11	15	50	0.434
II	0	2	3	2	7	
III	1	1	4	2	8	
IV	0	1	0	0	1	

Differences between groups tested by Pearson χ2 test for categorical and ANOVA for continuous variables. AR: Aortic regurgitation; CMRI: Cardiac Magnetic Resonance Imaging; AR0: patients without aortic regurgitation; AR1: patients with mild aortic regurgitation; AR2: patients with moderate aortic regurgitation; AR3: patients with severe aortic regurgitation; AV: AV; NYHA: New York heart association

**Table 2:** Echocardiographic and CMRI parameters for each AR grade as assessed by CMRI

	AR0 (*n* = 13)	AR1 (*n* = 16)	AR2 (*n* = 18)	AR3 (*n* = 19)	All patients (*n* = 66)	ANOVA
EFR (cm/s); *n* = 66	13.1 ± 0.9	13.2 ± 2.2	16.6 ± 4.4.5	20.8 ± 7.1*	16.3 ± 5.6	*p* < 0.001
PISA (mm); *n* = 66	0 ± 0	5.3 ± 1.6*	7.4 ± 1.*3	8.3 ± 1.6	5.7 ± 3.3	*p* < 0.001
EROA (mm²); *n* = 66	0 ± 0	0.2 ± 0.1*	0.1 ± 0.3*	0.3 ± 0.1	0.2 ± 0.1	*p* < 0.001
AR-Vol (ml); *n* = 66	0 ± 0	33.9 ± 20.3*	58.4 ± 26.2*	74.4 ± 26.1	45.6 ± 34.6	*p* < 0.001
VC (mm); *n* = 66	0 ± 0	4.3 ± 1.2*	5.1 ± 1.0	6.1 ± 1.6	4.2 ± 2.5	*p* < 0.001
PHT (ms); *n* = 53	n.a.	562 ± 144	415 ± 86*	350 ± 87	436 ± 137	*p* < 0.001
LV-EF (Echo) (%); *n* = 66	63 ± 7	58 ± 6	55 ± 13	56 ± 8	57 ± 8	*p* = 0. 118
LVEDD (echo, mm); *n* = 65	48 ± 5	53 ± 4	57 ± 7	62 ± 9	56 ± 8	*P* < 0.001
LVESD (echo, mm); *n* = 65	30 ± 7	34 ± 6	37 ± 10	38 ± 12	35 ± 10	*p* = 0. 116
LV-EF (CMRI; %); *n* = 66	60 ± 5	63 ± 6	56 ± 12	55 ± 12	58 ± 10	*p* = 0.106
LVEDV (CMRI; ml); *n* = 65	146 ± 27	196 ± 43	241 ± 64	261 ± 70	218 ± 70	*p* < 0.001
LVESV (CMRI; ml); *n* = 65	58 ± 11	73 ± 25	112 ± 55	122 ± 61	96 ± 52	*p* < 0.001

Data presented as mean values and standard deviations. Differences between groups tested with ANOVA. Post-Hoc analysis with Tukey test of echocardiographic parameters between groups (*=*p* < 0.05 between AR0- AR1, AR1-AR2 and AR2-AR3, respectively=). AR: Aortic regurgitation; AR0: patients without aortic regurgitation; AR1: patients with mild aortic regurgitation; AR2: patients with moderate aortic regurgitation; AR3: patients with severe aortic regurgitation; AR-Vol: regurgitant volume of aortic regurgitation; EFR: end diastolic flow reversal; EROA: effective regurgitation orifice area; LV-EF: left ventricular ejection fraction; LVESD: left ventricular end systolic diameter; LVEDV: left ventricular end diastolic volume; LVESV: left ventricular end systolic volume; CMRI: magnetic resonance imaging; PHT: pressure half time; PISA: proximal isovelocity surface area; VC: vena contracta

### Echocardiographic assessment of AR

The measurement of EFR showed high inter-observer and intra-observer agreement (intraclass correlation coefficient for inter-observer agreement 0.973 [95%-CI: 0.954–0.984] and for intra-observer agreement of 0.935 [95%-CI: 0.890–0.961]).

The overall echocardiographic AR grading correlated highly and significantly with CMRI-derived AR regurgitant fraction, which served as the reference for AR grading (Pearson correlation coefficient 0.893, *p* < 0.001).

The mean values of echocardiographic parameters used for AR assessment stratified by AR severity as determined by CMRI are shown in Table 2. The mean values of all traditional echocardiographic parameters - including PISA, EROA, AR-Vol, PHT as well as EFR - differed significantly between the four AR severity groups. Post-hoc analysis of the ANOVA revealed that EFR was the only parameter with a significant difference between severe and moderate AR (AR2 vs. AR3). In contrast, parameters obtained from the flow convergence method (PISA, EROA, and AR-Vol) showed significant differences between controls and mild (AR0 vs. AR1), as well as between mild and moderate AR (AR1 vs. AR2), but not between moderate and severe (AR2 vs. AR3). Similarly, VC measurements only differed significantly between mild and moderate AR in post-hoc testing (Table 2).

The sensitivity, specificity, PPV, and NPV for guideline-based thresholds of the echocardiographic AR parameters (EFR > 18 cm/s; PISA > 6 mm; EROA ≥ 30 mm²; AR-Vol ≥ 60 ml; VC > 6 mm; PHT < 200 ms) are shown in Table 3.

**Table 3:** Sensitivity, specificity, PPV and NPV for (semi-)quantitative echocardiographic parameters using established cutoffs

	Sensitivity	Specificity	PPV	NPV
EFR (cm/s); *n* = 66	57.9%	91.5%	73.3%	84.3%
PISA (mm); *n* = 59	84.2%	61.7%	47.1%	90.6%
EROA (mm²); *n* = 58	57.9%	85.1%	61.1%	83.3%
AR-Vol (ml); *n* = 59	73.7%	78.7%	58.3%	88.1%
VC (mm); *n* = 58	52.6%	83.0%	55.6%	81.3%
PHT (ms); *n* = 52	5.3%	100%	100%	72.3%

Echocardiographic parameter cutoffs (EFR > 18 cm/s; PISA > 6 mm; EROA ≥ 30 mm²; AR-Vol ≥ 60 ml; VC > 6 mm; PHT < 200 m). AR-Vol: regurgitant volume of aortic regurgitation; EFR: end diastolic flow reversal; EROA: effective regurgitation orifice area; NPV: negative predictive value; PHT: pressure half time; PISA: proximal isovelocity surface area; PPV: positive predictive value; VC: vena contracta

**Fig. 2:**
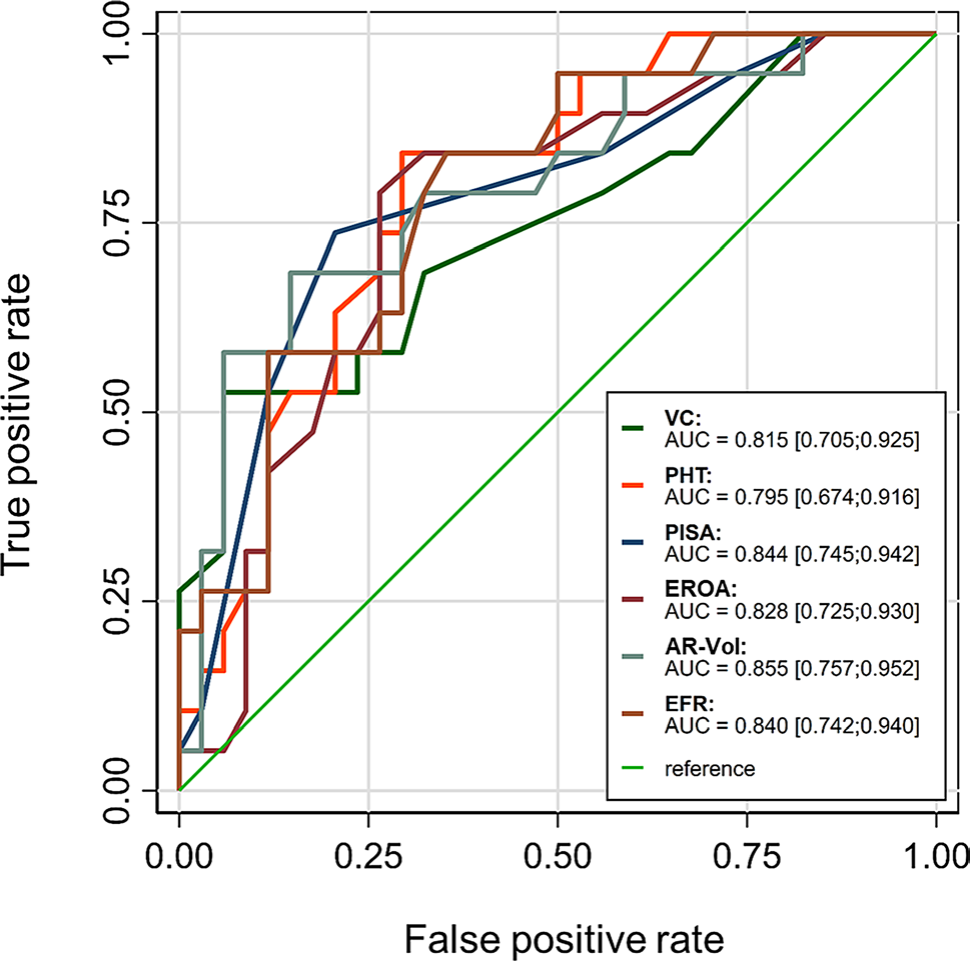
Receiver operating characteristics curves showing diagnostic performance of AR echocardiographic parameters. Diagnostic value of selected echocardiographic parameters for diagnosing severe AR in comparison to CMRI (EFR, PISA, EROA, AR-Vol, VC and distinguishing severity of AR (PHT distinguishing severe from mild and moderate AR). AR: aortic regurgitation; AR-Vol: regurgitant volume of aortic regurgitation; AUC: area under the curve; EFR: end diastolic flow reversal; PHT: pressure half time; PISA: proximal isovelocity surface area; ROC: receiver operating characteristics; VC: vena contracta

The diagnostic accuracy of echocardiographic parameters for diagnosing the severity of AR in comparison to CMRI was further analyzed using ROC curves. All individual parameters demonstrated good diagnostic performance (AUC for EFR: 0.84 ± 0.05; AUC for VC: 0.82 ± 0.06; AUC for PISA: 0.84 ± 0.05; AUC for EROA: 0.83 ± 0.05; AUC for AR-Vol: 0.86 ± 0.05; *p* < 0.001 for all parameters, same results with original and z-transformed values; Fig. 2a).

Since PHT could only be measured in the presence of AR, its diagnostic accuracy was evaluated by distinguishing between different AR severities. PHT demonstrated good accuracy (AUC: 0.79 ± 0.06 for differentiating severe from mild and moderate AR, *p* < 0.001; Fig. 2b).

### Comparison of echocardiographic parameters of AR

Pairwise comparisons of AUC values of the traditional echocardiographic markers of severe AR and EFR did not show significant differences (VC vs. EFR: *p* = 0.70; PISA vs. EFR: *p* = 0.94; EROA vs. EFR: *p* = 0.79; AR-Vol vs. EFR: *p* = 0.79 for diagnosing severe AR; PHT vs. EFR: p 0.98 for differentiating between AR severities).

The potential relevance of EFR compared to traditional echocardiographic parameters was further assessed with a multivariable logistic regression model with backward variable selection. Using an initial model with EFR, PISA, EROA, AR-Vol, and VC as continuous variables, only EFR and VC remained significantly associated with severe AR as determined by CMRI (model *p* < 0001, VC Odds Ratio [OR] 2.11 [95%-CI: 1.17–3.83] *p* = 0.013, EFR OR 1.19 [95%CI: 1.03–1.28] *p* = 0.022).

A further second logistic regression model was performed using the same five parameters as categorical variables with guideline-based cutoffs (PISA > 6 mm; EROA ≥ 30 mm²; AR-Vol ≥ 60 ml; VC > 6 mm; EFR > 18 cm/s). In this model, EFR, AR-Vol, and VC were significant predictors of severe AR (EFR: *p* < 0.001, EFR OR 6.92 [95%-CI:1,36-35.35] *p* = 0.020, AI-vol OR 3.56 [95%-CI:0.80-15.78] *p* = 0.095, VC OR 3.73 *p* = 0.073 [95%-CI: 0.89–12.66].

### Clinical endpoints and correlation

Over a mean follow-up period of 62 months [range: 30–80], 16 of the 53 patients (24.2%) underwent AV surgery, while 4 (6.1%) were lost to follow-up. None of the controls developed severe AR requiring surgery.

Except for PHT, all echocardiographic parameters were significantly different between patients who underwent AV surgery and those not needing surgery when analysed as continuous variables (*p* < 0.001 for PISA, EROA, AR-Vol and VC, *p* = 0.043 for EFR, *p* = 0.41 for PHT, Mann-Whitney-U-test). Significantly more patients receiving AV surgery showed values above the established cutoffs of VC and flow convergence-derived measurements (PISA, EROA, AR-Vol) compared to patients without the need for surgery (*p* < 0.001 for PISA; *p* = 0.046 for EROA; *p* = 0.005 for AR-Vol; *p* = 0.018 for VC; Pearson χ^2^ test). EFR > 18 cm/s showed a trend (*p* = 0.061). In contrast this was not the case for PHT (*p* = 0.47).

**Fig. 3:**
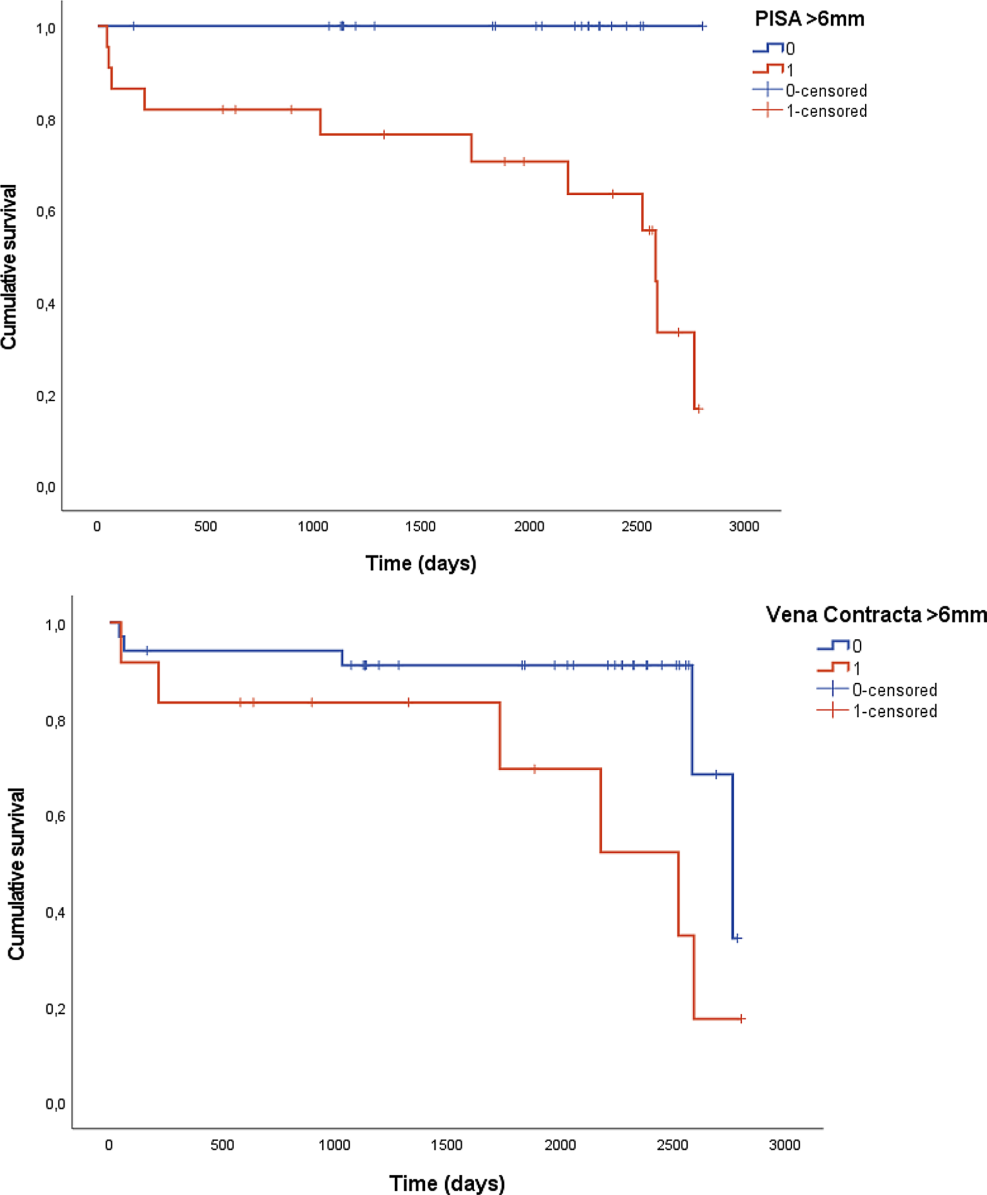
Differences in time to AV surgery according to the echocardiographic parameters VC and PISA (Kaplan-Meier curves and Log-Rank tests, *p* = 0.002 for PISA, *p* = 0.040 for VC). VC: Vena Contracta, PISA: Proximal Isovelocity Surface Area

Kaplan-Meier curves for time to surgery and the Log-Rank tests demonstrated significant differences for PISA (*p* = 0.002) and VC (*p* = 0.040, Fig. 3), but not for other parameters (*p* = 0.29 for EROA; *p* = 0.09 for AR-Vol; *p* = 0.47 for EFR).

Cox-regression analysis including the five echocardiographic variables (EFR, PISA, EROA, AR-Vol, VC) using a backward variable selection with a conservative cut-off of *p* = 0.157 retained only VC as being significantly associated with time to AV surgery (*p* = 0.024, HR = 1.56 [95%-CI: 1.06–2.29]).

## Discussion

In clinical practice accurate evaluation of chronic AR severity- particularly distinguishing between moderate and severe AR- is crucial for appropriate clinical decision-making and for determining the optimal timing of surgery before irreversible cardiac damage occurs [2, 20–22]. The EACVI, ASE and BSE recommend the inclusion of EFR-measurement in the stratification of AR severity [3, 14]. However, it remains unclear whether this parameter improves the diagnostic accuracy of echocardiographic AR grading and the prediction of the need for AV surgery. The results of this prospective study provide important insights into the relevance of several echocardiographic parameters, including EFR, for assessing AR in comparison to CMRI, as well as the prognostic value of these parameters for predicting the need for valve surgery in patients with chronic AR during mid-term follow-up:

1. Compared to the reference of CMRI-derived AR regurgitant fraction, all echocardiographic parameters demonstrated good diagnostic value in grading AR severity. However, in in a logistic regression analysis model using continuous values, only EFR and VC were retained as significant predictors of AR severity. This suggests that these two echocardiographic parameters have diagnostic relevance.

2. EFR, with a cut-off of > 18 cm/s, showed moderate sensitivity (57,9%) and high specificity (91,5%) in grading aortic insufficiency as severe.

3. Furthermore, all echocardiographic parameters, except PHT, showed differences between patients receiving AV surgery and those not receiving AV surgery. However, only VC was associated with time to AV surgery.

To our knowledge, our study is one of the first to analyze the potential predictive value of echocardiographic EFR velocity in determining the need for AV surgery in patients with chronic AR, compared to traditional echocardiographic parameters.

### Correlation of echocardiographic AR quantification parameters with CMRI

The primary aim of this study was to evaluate classical echocardiographic parameters, as well as EFR, for assessing AR severity in comparison to the reference standard, CMRI. In addition to direct echocardiographic criteria, an indirect sign of severe AR is LV dilatation, and, in very advanced cases, impaired LV function. In our cohort, mean LV-EF, measured by both echocardiography and CMRI, was preserved (>55%) across all AR grades. This aligns with the findings of Tarasoutchi et al. and Messika-Zeitoun et al., who observed that LV function deteriorates only late in advanced AR [23, 24]. On the other hand, LV dimensions and volumes, such as LVEDD on echocardiography and LV volume on CMRI, are often enlarged in severe AR. This was also the case in our study and in other prospective studies [25]. Furthermore, LV dilatation seems to be of prognostic relevance. Yang et al. found that LV end-systolic dimension index was the only LV parameter independently associated with all-cause mortality in a large cohort of patients with moderate and severe AR receiving either surgery or medical treatment [22].

In our study, all classic echocardiographic parameters recommended by echocardiographic guidelines for AR grading (VC, PHT and PISA-derived parameters as EROA and AR-Vol) [3, 14], as well as the newer parameter EFR, provided good diagnostic value for diagnosing severe AR as determined by CMRI (AUC ≥ 0.79 for all mentioned parameters). Individual comparison of the AUC for classical echocardiographic parameters versus EFR showed no significant differences, suggesting that EFR is at least as valid as VC, PHT and PISA-derived values for grading AR.

Moreover, using a logistic regression model, only EFR and VC were retained as significantly being associated with severe AR, suggesting that these parameters may improve diagnostic value compared to traditional echocardiographic parameters. This is consistent with the literature: Hlubocka et al. found the best correlation between indirect echo-Doppler parameters and CMRI parameters for EFR and 3D Vena contracta [26].

As expected, the mean values of all echocardiographic parameters, including EFR-velocity, increased with increasing AR-severity. However, mean EFR velocities differed significantly between moderate and severe AR, which was not the case for the traditional echocardiographic parameters, where the means were only significantly different between mild and moderate or none and mild AR. Since differentiating moderate from severe AR is the most challenging but also most clinically relevant, this finding highlights the importance of integrating EFR in the echocardiographic assessment of AR. In summary, our findings support the ESC, EACVI and BSE echocardiography guidelines [2–4, 14], which recommend using all echocardiographic AR parameters for comprehensive evaluation of AR severity. Concordantly, a study by Gao et al. demonstrated the utility of EFR in accurately identifying or excluding severe chronic aortic regurgitation, using CMRI as the reference standard [27]. EFR, with a cut-off of >18 cm/s, showed moderate sensitivity (57,9%) and high specificity (91,5%) in grading aortic insufficiency as severe. Similar results were found by Messika-Zeitoun et al. [23], Gao et al. [27], and Beck-Hansen, who even showed an improvement in diagnostic accuracy with even lower thresholds (>17 cm/s and >13 cm/s respectively) [28], although these studies did not use clinical endpoints. Moreover, although both EFR and PHT reflect diastolic backflow dynamics and are influenced by aortic compliance, EFR demonstrated superior diagnostic performance. In contrast, PHT, despite its high specificity in this study, was limited by low sensitivity. The reliance of both parameters on aortic compliance may contribute to measurement variability, highlighting the need for future studies to assess the impact of vascular properties on their reliability [29].

Low intra- or inter-observer variability in our EFR-measurements confirms that EFR is highly reproducible. Interestingly, EFR velocity correctly classified AR grade in 82% of the patients. Therefore, since EFR is a robust and reliable parameter measured distal from the AV from the suprasternal view, it could serve as a helpful additional parameter in AR grading in patients in whom traditional parameters at the AV level may be difficult to obtain, such as in cases of significant AV calcification, AV replacements, and subsequent artifacts from shadowing, or very eccentric jets. This is also emphasized in a study by Hlubocka et al., which found that the feasibility of measuring the PISA parameters was low, as they were only measurable in about 50% of patients [26].

Other EFR parameters also appear promising and should be further evaluated in prospective clinical studies, such as the diastolic velocity time integral, which has been shown to be superior to pulsed-wave EFR [28], and holodiastolic flow reversal in the abdominal aorta, measured from a subcostal window, which is highly sensitive (100%) and specific (97%) for severe AR [30, 31]. However, further studies evaluating these promising EFR-derived parameters are needed.

### Echocardiographic AR quantification parameters for prediction of AV surgery

In our study, all echocardiographic parameters (PISA, EROA, AR-Vol, VC and EFR), except PHT, were significantly different between patients receiving AV surgery compared to patients not needing surgery. However, in a Cox regression analysis including the five echocardiographic variables (EFR, PISA, EROA, AR-Vol, VC), only VC was retained as predictive of time to AV surgery. Similarly, another clinical outcome study by Kočková et al. examining new imaging markers in patients with asymptomatic severe chronic AR, found that 3D Echo-derived vena contracta area and CMRI-derived regurgitant volume and fraction had high accuracy and excellent sensitivity in identifying patients in need of early surgery. In their study, EFR with a cutoff of 22 cm/s showed a moderate sensitivity (65%) and moderate to high specificity (78%) in predicting patients who would undergo AV surgery [25]. Our findings also align with Attar et al. (2025) [32], who also reported VC as a leading echocardiographic marker in comparison to CMRI. Kammerlander et al. [33] found CMR-derived RF and NT-proBNP levels as strongest predictors of adverse outcomes, emphasizing the importance of multimodal evaluation.

## Limitations

There are several potential limitations to our study. The small sample size with consecutively limited event rates in the survival analysis might lead to risk of model overfitting. Besides, exclusion of certain patient subgroups may limit generalizability. Additionally, patients in whom EFR was not measured were excluded from our study. Therefore, this study should not be considered a feasibility study. We also excluded patients with severe concomitant valvular or cardiac abnormalities and atrial fibrillation, so our patient population may not represent the entire spectrum of AR patients, which may limit generalizability. However, the patients included in our study had a wide range of ages and symptoms.

Furthermore, the study’s endpoint (AVR) may introduce incorporation bias, as echocardiographic and CMRI findings guide surgical referral. However, the decision to proceed with AV surgery is based on the clinical judgement of the treating physician, primarily guided by disease severity, imaging and clinical parameters, in line with clinical practice. CMR regurgitant fraction, used as the reference standard in this study, is justified given its reproducibility and endorsement in current guidelines, particularly when echocardiographic assessment is inconclusive. However, it is not without limitations. While we applied established thresholds (>40%) to define severe AR, *though data suggesting lower thresholds (32–33%) are emerging* [34, 35].

Finally, there are methodical limitations of the individual echocardiographic parameters assessing AR, challenging direct comparison. For example, assessment of PHT is only possible if AR is present. Finally, we acknowledge that our results are exploratory and thus should to be interpreted with caution and validated in a larger cohort with more events that avoids overfitting in the modeling process. Especially, larger, multicenter studies using hard endpoints, like mortality or heart failure hospitalization, are warranted.

## Conclusions

EFR provides added value in AR severity assessment, especially for distinguishing moderate from severe regurgitation. However, VC was the strongest echocardiographic predictor of AV surgery. A comprehensive assessment integrating EFR, VC, and standard echocardiographic parameters should be pursued, supported by CMR when indicated.

## Electronic Supplementary Material

Below is the link to the electronic supplementary material.

Supplementary Material 1

## Data Availability

The datasets used and/or analysed during the current study are available from the corresponding author on reasonable request.

## Abbreviations

ANOVA: Analysis of variance
AR: Aortic regurgitation
AR0: Patients without aortic regurgitation
AR1: Patients with mild aortic regurgitation
AR2: Patients with moderate aortic regurgitation
AR3: Patients with severe aortic regurgitation
AR-Vol: Regurgitant volume of aortic regurgitation
ASE: American Society of Echocardiography
AUC: Area under the curve
AV: Aortic valve
BSE: British Society of Echocardiography
CMRI: Cardiac magnetic resonance imaging
EACVI: European Association of Cardiovascular Imaging
EFR: End diastolic flow reversal
EROA: Effective regurgitation orifice area
ESC: European society of cardiology
LV: Left ventricle / left ventricular
LV-EF: Left ventricular ejection fraction
LVESD: Left ventricular end systolic diameter
LVEDV: Left ventricular end diastolic volume
LVESV: Left ventricular end systolic volume
NPV: Negative predictive value
PHT: Pressure half time
PPV: Positive predictive value
PISA: Proximal isovelocity surface area
RF: Regurgitant fraction
ROC: Receiver operating characteristics
SD: Standard deviation
VC: Vena contracta
