# Supplementary material for: Assessment of chronic aortic regurgitation using end-diastolic flow reversal in the upper descending aorta: diagnostic accuracy and prediction of aortic valve surgery in a prospective echocardiography and cardiac magnetic resonance imaging study
Source: Echo Res Pract. 2026 Jan 5;13:1. doi: 10.1186/s44156-025-00101-3 (PMC12766934; doi:10.1186/s44156-025-00101-3)
Supplement: Supplementary file 1 — Supplementary Material 1 [file 44156_2025_101_MOESM1_ESM.docx]

**Supplement**


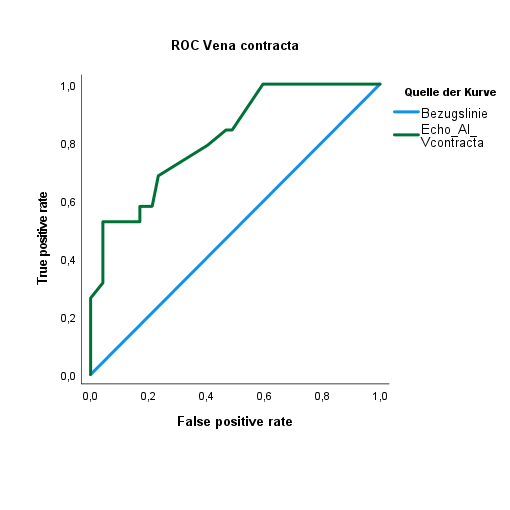

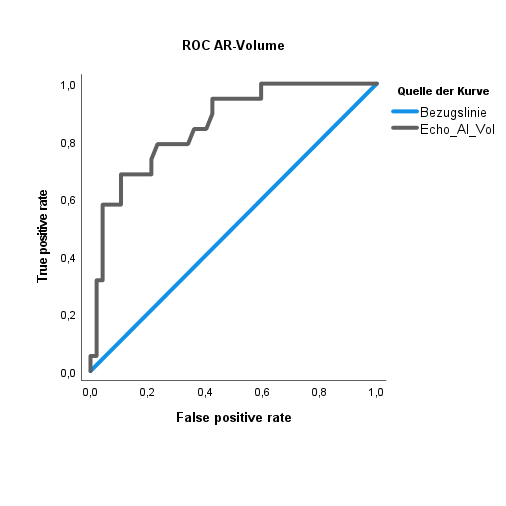

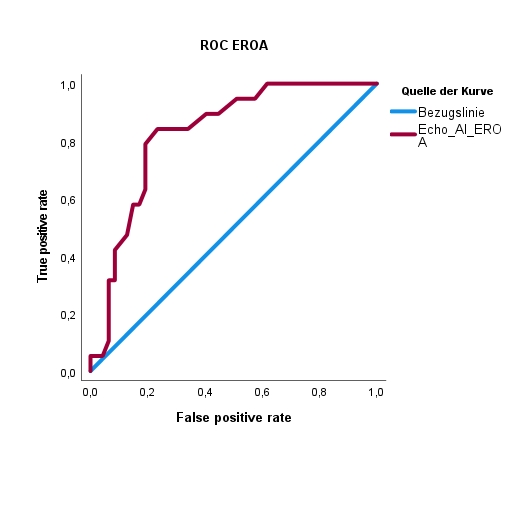

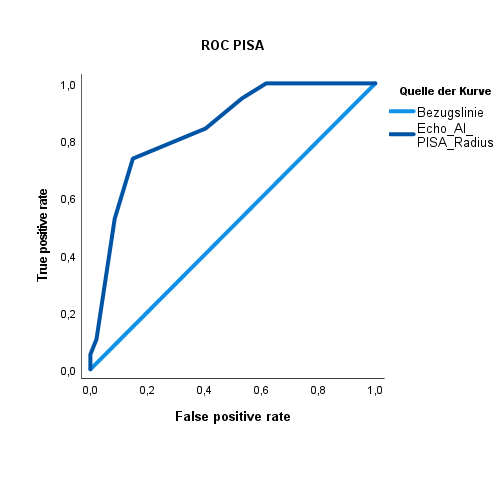

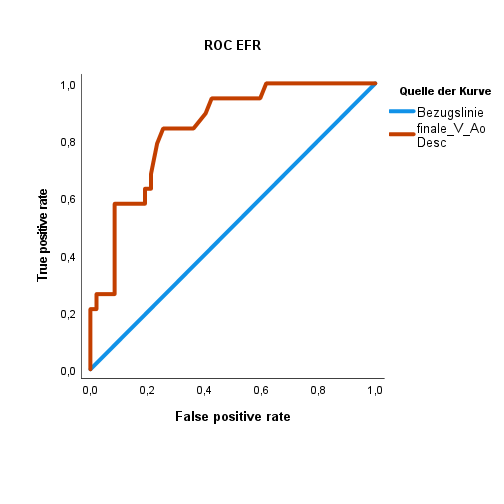

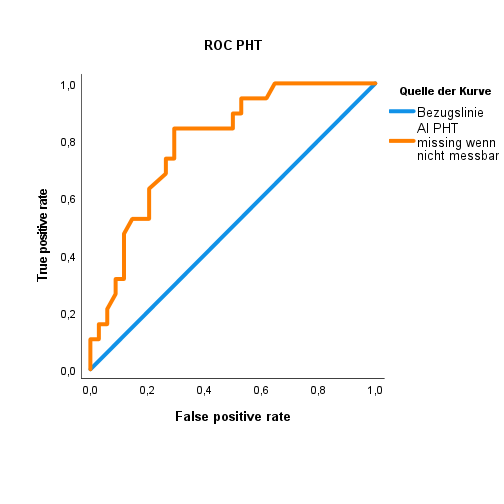


**Supplemental figure 1: Individual receiver operating characteristics curves showing diagnostic performance of AR echocardiographic parameters individually**

Diagnostic value of selected echocardiographic parameters for diagnosing severe AR in comparison to CMRI (EFR, PISA, EROA, AR-Vol, VC and distinguishing severity of AR (PHT distinguishing severe from mild and moderate AR).
